# Comparative study on complications of screws versus plates for comminuted radial head and neck fractures with two or three fragments

**DOI:** 10.3389/fsurg.2025.1731596

**Published:** 2026-01-15

**Authors:** Yuliang Fu, Yuan Cao, Zengzhen Cui, Liangyu Bai, Xiaoyu Norman Pan, Yang Lv

**Affiliations:** 1 Department of Orthopedics, Peking University Third Hospital, Beijing, China; 2 Engineering Research Center of Bone and Joint Precision Medicine, Beijing, China; 3 Department of Physical Medicine and Rehabilitation, Harvard Medical School/Spaulding Rehabilitation Hospital, Boston, MA, United States; 4 Department of Medicine, MetroWest Medical Center, Framingham, MA, United States

**Keywords:** headless compression screw, open reduction and internal fixation, plate, radial head fracture, radial neck fracture

## Abstract

**Background:**

This retrospective study aimed to compare the efficacy of internal fixation using headless compression screws (HCS) and radial head locking plate (RLP) for comminuted radial head and neck fractures with no more than 3 displaced fragments.

**Methods:**

This retrospective study included 84 patients with radial head and neck fractures treated at Peking University Third Hospital between January 2013 and December 2022, with 38 and 46 patients in the HCS and RLP groups, respectively. The main outcome was the comparison of complications between the two groups. Demographic data, pre-operative time (POT), operation time (OT), and hospital stay time (HST) were also recorded. The Mayo Elbow Performance Score (MEPS), range of movement of the elbow and forearm, and reasons for re-operation were compared between the two groups.

**Results:**

All patients were followed up for an average of 66.4 months (range, 20–135 months). One patient in each group underwent radial head replacement due to non-union, while the remaining patients achieved bone union. There was no statistically significant difference in the clinical outcomes between the two groups (*p* > 0.0023). Additionally, the re-operation rate due to symptomatic hardware was significantly higher in the RLP group (28.3%) than that in the HCS group (2.6%, *p* = 0.002).

**Conclusion:**

For internal fixation of comminuted radial head and neck fractures with no more than three displaced fragments, both HCS and RLP achieved good outcomes. However, the RLP increased the incidence of complications and re-operation associated with internal fixation compared to HCS.

## Background

The radial head plays a crucial role in the elbow joint stability because approximately 60% of the longitudinal load on the elbow is transmitted through the radiocapitellar joint. Radial head fractures account for 1.7%–5.4% of all fractures, usually caused by indirect trauma (1). Axial and valgus forces cause the anterolateral radial head to impact the capitellum, leading to fracture (2). It may lead to elbow instability, stiffness, chronic pain, and other complications if not managed (2). Therefore, the management of acute radial head fracture is crucial.

Mason type Ⅰ fractures, which include a fissure or marginal sector fracture without displacement, can be treated non-operatively with early mobilization (3, 4). For displaced or comminuted radial head fractures, non-operative management may result in complications such as elbow stiffness and fracture non-union (5). Therefore, surgical interventions, such as radial head excision, open reduction and internal fixation (ORIF), and radial head replacement have become the mainstay of treatment for comminuted radial head fractures in recent decades (5–7). The radial head replacement is mainly suitable for patients with complete articular radial head fractures with more than three displaced fragments (7). Furthermore, resection may lead to perceived elbow instability in patients compared with ORIF, thus it is more commonly used for elderly people with lower functional requirements (6). For young and middle-aged patients with comminuted radial head and neck fractures with no more than 3 displaced fracture fragments, ORIF is often the first strategy (6).

ORIF methods include screws, plates and Kirschner wires (K-wires). Smith et al (8) proposed that at least two oblique cross-screws should be used to fix radial head and neck fractures. Therefore, more screws are required when radial head fractures are combined with neck fractures, which increases the risk of screw interference and surgical difficulty. Gruszka D et al (9) treated complex radial head neck fractures with locking compression plates, showing a mean MEPS of 90 points with no poor outcomes. Available evidence suggests no consensus on the optimal fixation method, as each fixation has distinct clinical advantages.

Current research shows limited clinical comparisons of surgical approaches for radial head neck fractures. Therefore, comparative clinical studies are necessary. Our retrospective cohort study compared the complications on headless compression screws (HCS) and radial head locking plates (RLP) in treating comminuted radial head and neck fractures with two or three fragments.

## Methods

This retrospective study was complied with the principles of the Declaration of Helsinki and approved by the ethics committee of Peking University Third Hospital (Approval Number: IRB00006761-M20250573). Written informed consent was obtained from each participant before commencement.

### Design and population

**Figure 1:**
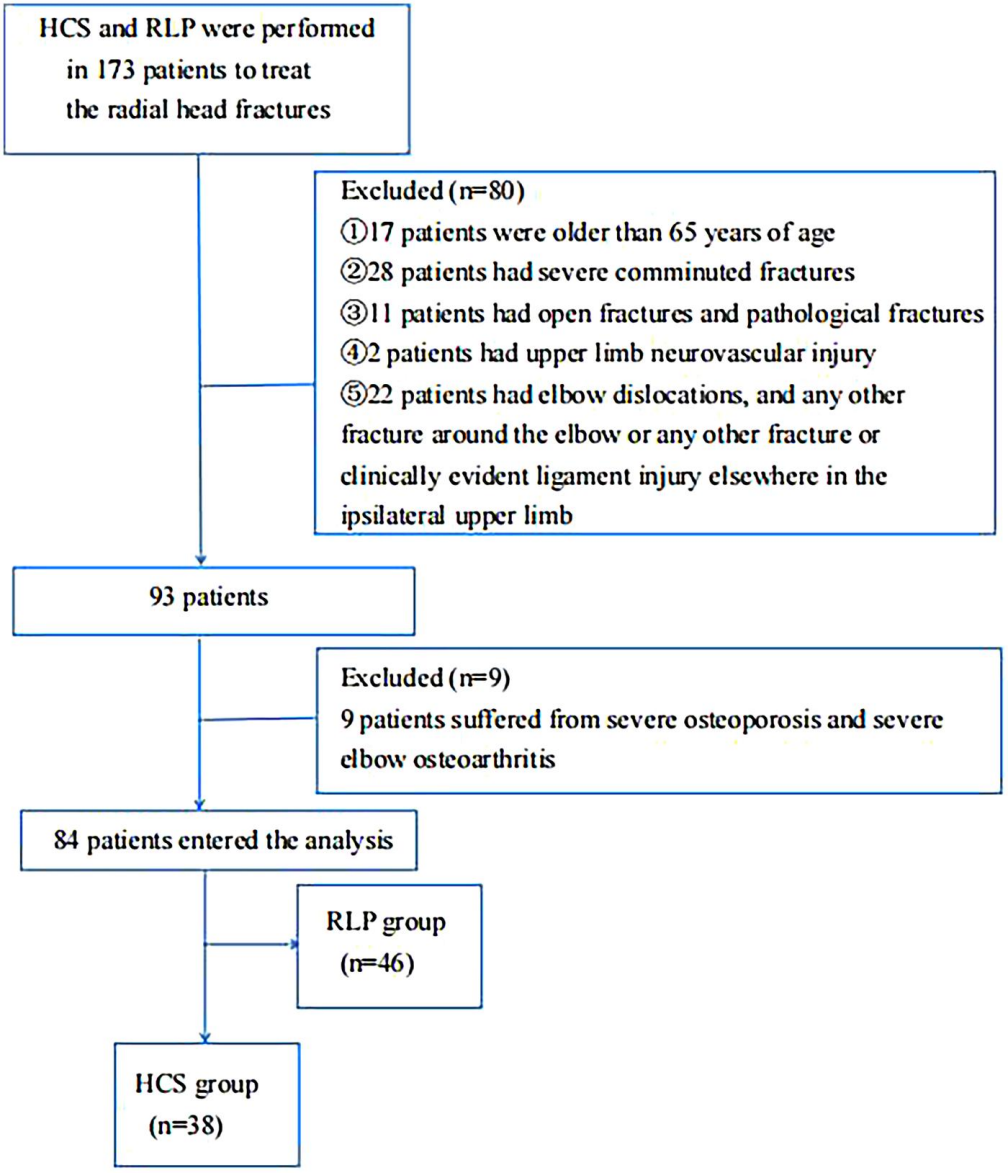
A flow diagram of the research.

Between January 2013 to December 2022, 173 patients with fractures of the radial head were surgically and retrospectively evaluated. After verifying the inclusion and exclusion criteria, 84 patients (48.6%) were included (Figure 1). Fracture types and radial head fracture fragments were determined based on the 3-dimensional (3D) reconstruction of elbow joint computed tomography (CT) scans.

The main inclusion criteria were: (1) age of 18–65 years, (2)acute unilateral closed injury (<2 weeks), and (3) radial neck fractures with displaced radial head fragmenting into two or three piece. Exclusion criteria were: (1) severe comminuted fractures (radial head fracture fragments >3), (2) open fractures and pathological fractures, (3) elbow dislocations, and any other fracture around the elbow or any other fracture or clinically evident ligament injury elsewhere in the ipsilateral upper limb, (4) concomitant upper limb neurovascular injury affecting post-operative evaluation, and (5) severe osteoporosis and severe elbow osteoarthritis.

### Surgical strategy

Patients were divided into the HCS group and the RLP group based on surgeon preference for fixation with either HCS or RLP. Under ultrasound-guided brachial plexus block anaesthesia, both groups were placed in a horizontal position using tourniquets. The Kocher approach was used in all patients. The fracture was exposed, and the fracture fragments were examined. After identifying fracture fragments, radial head articular pieces without soft tissue attachments were removed. Fracture reduction was done with clamps or Kirschner wires (K-wires). In cases of completely detached fragments, radial head reconstruction was performed on the surgical table using the “on-table reconstruction” technique (10).

**Figure 2:**
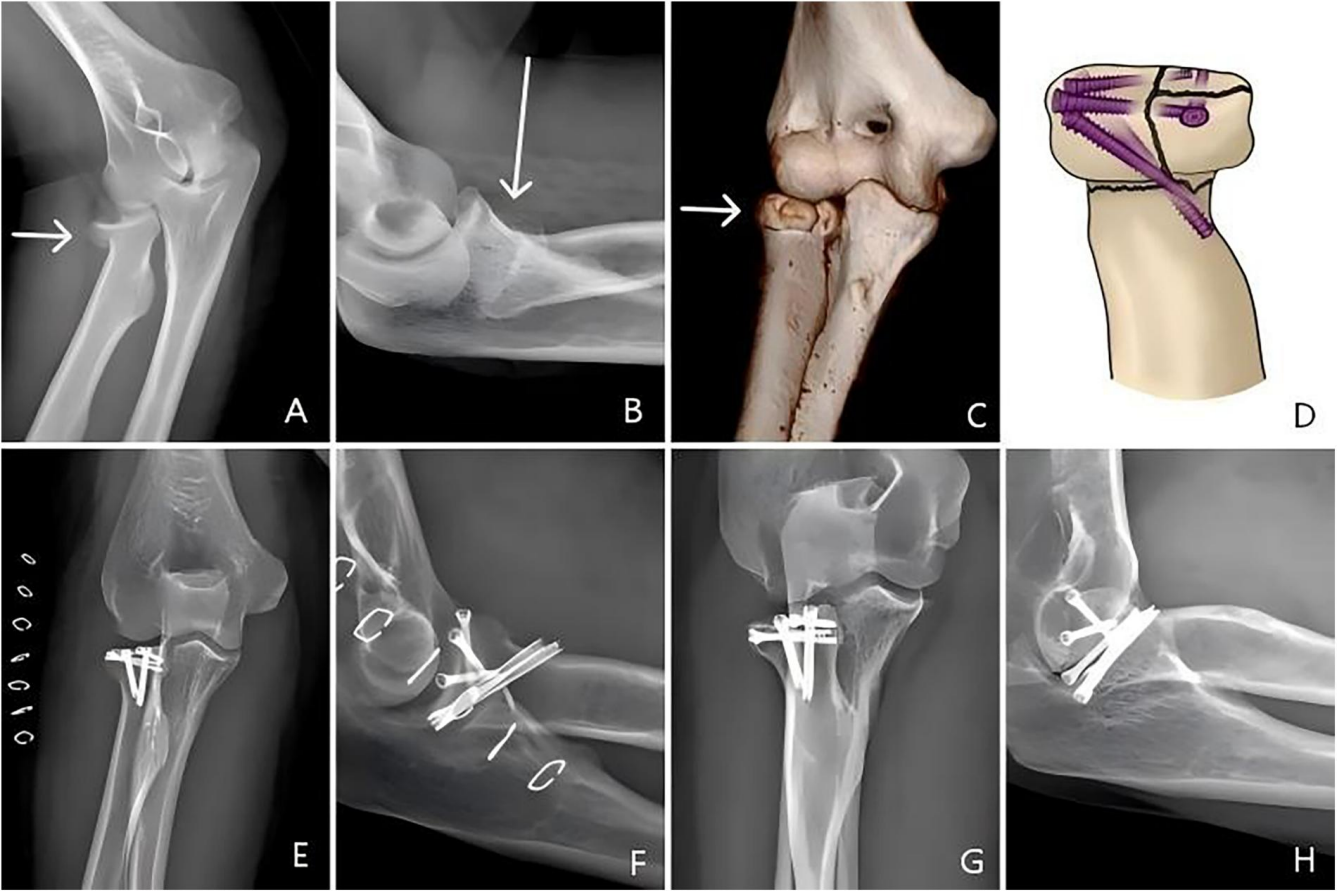
Headless compression screw fixation: **(A–C)** Pre-operative AP/lateral views and 3D CT reconstruction showing radial head neck fracture with three comminuted fragments; **(D)** fixation diagram using transverse screw combined with oblique crossed screws; **(E,F)** 1-day post-operative radiographs; **(G,H)** 18-month follow-up radiographs confirming fracture union.

In the HCS group, HCSs were placed in an selected position to achieve fragment fixation (Figure 2). Post-reduction stabilisation required: (1) at least one transverse screw per fragment providing interfragmentary compression, and (2) a minimum of two intercrossing screws engaging the radial shaft cortex. All screw heads were recessed 1–2 mm below the subchondral bone margin to prevent articular surface protrusion.

**Figure 3:**
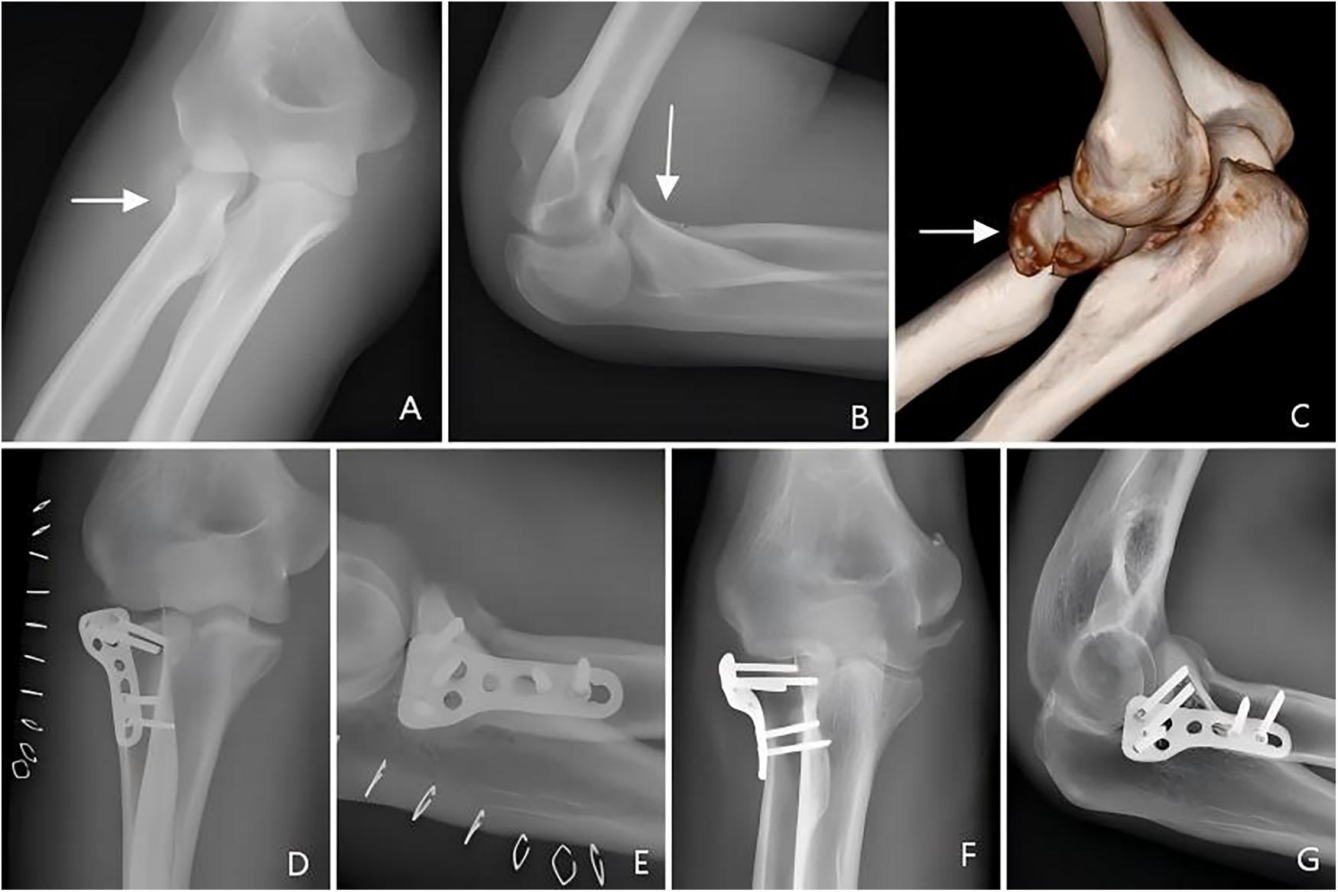
Radial head locking plate fixation: **(A–C)** Pre-operative AP/lateral views and 3D CT reconstruction showing radial head neck fracture with two fragments; **(D,E)** 1-day post-operative radiographs; **(F,G)** 15-month follow-up radiographs confirming fracture union.

In the RLP group, fixation was achieved using a pre-contoured anatomic RLP with three proximal and two distal multidirectional locking screws. Full forearm rotation was verified intra-operatively to ensure the radial head was within the safe zone. The safe zone referred to the area on the radial head that did not participate in the proximal radioulnar joint (9) (Figure 3).

After fixation, stability was verified through passive elbow movement and unrestricted forearm rotation. The wound was irrigated and closed in layers, and post-operative bracing was applied in a neutral position.

### Postoperative care and outcome measurement

Discharge required stable vitals, no infection signs. All patients followed a similar rehabilitation protocol. Radiographic evaluation was performed in all patients with anteroposterior and lateral elbow radiographs at 2 weeks, 1, 2, 3, 6, 12 months and the last follow-up postoperatively. X-rays at 2 weeks confirmed implant stability before starting range of motion exercises. Weight bearing was not allowed for 4 weeks in all patients. Weight-bearing rehabilitation was gradually escalated based on radiographic evidence of fracture consolidation, while early overloading was strictly avoided. Bone union was defined by a complete fracture line blurred with continuous callus formation and pain-free weight-bearing capacity of the elbow joint.

The following parameters were collected from both groups: pre-operative time (POT, time from initial injury to surgery), operation time (OT), and hospital stay time (HST, time from surgery to discharge). The evaluation was conducted by comparing the Mayo Elbow Performance Score (MEPS) (11), range of movement of the elbow and forearm. Post-operative complications and reasons for re-operation were also observed.

Clinical outcome evaluation was performed in all patients at the last follow-up using the MEPS and assessing elbow range of movement. The MEPS was classified as excellent (>90), good (75–89), fair (60–74), and poor (<60). Postoperative functional outcomes (MEPS, ROM) were evaluated by an independent assessor who was blinded to the fixation method used.

Post-operative complications and the reasons for secondary surgery were also assessed at the last follow-up. Complications necessitating surgical treatment were considered major complications, while all others were classified as minor. The radiographic assessment of heterotopic ossification (HO) was performed by two clinicians using the Hastings-Graham classification system. Diagnosis of posterior interosseous nerve (PIN) injury was based on clinical symptoms and confirmed by electromyography (EMG). Recovery was defined as the return of muscle strength to MRC grade 4 or above, accompanied by the resolution of sensory symptoms.

### Data analysis

Data were analysed using SPSS 27.0 statistical software (IBM Corp, Armonk, NY, USA). Data are reported as Mean ± SD unless otherwise noted. The normality of continuous variables was tested using the Shapiro–Wilk test. Parametric tests (two-tailed independent *t*-tests) were used for normally distributed data; otherwise, the Mann–Whitney *U*-test was applied. Categorical variables were analysed using the chi-square test or Fisher's exact test. To account for multiple comparisons (21 hypothesis tests), adjusted *p*-values were calculated using the Bonferroni correction. Statistical significance was set at *p* < 0.0023. To control for potential confounding factors, we performed multivariable logistic regression analyses for key outcomes (Tables 1, 2): total complications and major complications requiring surgical intervention. The models were adjusted for the following pre-specified covariates: fixation method (HCS group vs. RLP group), age (continuous), sex (male vs. female), number of radial head fragments (2 vs. 3) and calendar year of surgery. The results are presented as adjusted odds ratios (aOR) with 95% confidence intervals (CI).

**Table 1:** Multivariable logistic regression analysis for total complications.

Variable	Adjusted OR (95% CI)	*P*-value
Fixation method	5.20 (1.74–15.52)	**0** **.** **003**
Age	1.01 (0.97–1.06)	0.675
Sex	1.52 (0.49–4.68)	0.466
Fragment number	2.54 (0.88–7.37)	0.086
Calendar year	1.09 (0.90–1.33)	0.380

*P*-values in bold denote statistical significance at the *P* < 0.05 level.

**Table 2:** Multivariable logistic regression analysis for major complications requiring surgery.

Variable	Adjusted OR (95% CI)	*P*-value
Fixation method	9.22 (1.87–45.41)	**0** **.** **006**
Age	1.03 (0.97–1.08)	0.343
Sex	1.58 (0.41–6.04)	0.506
Fragment number	2.80 (0.77–10.19)	0.118
Calendar year	0.98 (0.78–1.24)	0.886

*P*-values in bold denote statistical significance at the *P* < 0.05 level.

### Sensitivity analysis

To assess the robustness of our findings, a sensitivity analysis was conducted using worst-case imputation for the two cases of fixation failure (one in each group) that were initially excluded from functional outcome analyses. For these patients, we imputed the worst plausible values for time to union (set as the maximum time limit for the diagnosis of non-union fractures), MEPS (set as the minimum possible score of 0), and range of motion (set as the worst angle for all movements). The imputed data were then included in all outcome analyses.

## Results

### Group characteristics

The mean follow-up period was 67.2 ± 32.9 (range 20–135) months. In total, 84 patients with unilateral radial head and neck fractures were included. The HCS group included 38 patients (15 males and 23 females, mean age 37.8, range 20–64 years), who underwent treatment with the HCS (Figure 1). The RLP group included 46 patients (19 males and 27 females, mean age 37.1, range 20–63 years) who underwent treatment with the RLP (Figure 2). There were no significant differences between the two groups in terms of demographics, radial head fragment number, POT, OT, or HST (Table 3). All SMDs were <0.1 except for OT and HST, which were <0.3, indicating an acceptable balance of baseline characteristics between the two groups.*

**Table 3:** Comparison of the patient characteristics of the HCS group and RLP group.

Characteristic	HCS group *n* = 38	RLP group *n* = 46	SMD	*P*-value
Age (years)	37.8 ± 13.2	37.1 ± 11.6	0.06	0.822
Sex (M/F)	15/23	19/27	0.03	0.865
Radial head fragment number 2/3	24/14	26/20	0.11	0.537
Fracture side (R/L)	20/18	22/24	0.07	0.661
Dominant side (R/L)	34/4	42/4	0.09	0.776
follow-up time (months)	66.7 ± 31.9	67.7 ± 34.0	0.02	0.929
POT (days)	4.2 ± 3.2	3.9 ± 2.4	0.11	0.858
OT (min)	60.9 ± 27.3	69.3 ± 38.0	0.25	0.314
HST (days)	2.4 ± 1.0	2.7 ± 1.6	0.22	0.428

POT, preoperation time; OT, operation time; HST, hospital stay time; R, right; L, left; M, male; F, female; HCS, headless compression screw; RLP, radial head locking plate; SMD, standardized mean difference.

### Clinical outcome

Based on follow-up evaluations, one patient in each group underwent radial head replacement due to non-union, while the remaining patients achieved bone union. Both fixation methods demonstrated favourable functional recovery, with no statistically significant differences in time to union or MEPS. Additionally, there were no significant differences in the range of movement of the elbow joint between the groups, including flexion, extension deficit, and forearm rotation (Table 4).

**Table 4:** Postoperative follow-up data.

Outcome	HCS group *n* = 38	RLP group *n* = 46	SMD	*P*-value
Time to union (week)	14.0 ± 6.7	14.6 ± 6.3	−0.09	0.145
MEPS	94.7 ± 16.7	93.8 ± 15.6	0.06	0.394
Range of movement				
Flexion (°)	126.8 ± 8.2	128.0 ± 6.8	−0.16	0.577
Extension (°)	7.3 ± 15.9	7.2 ± 14.6	0.01	0.386
Pronation (°)	76.9 ± 14.0	76.9 ± 15.9	0.00	0.205
Supination (°)	76.1 ± 13.6	75.0 ± 12.2	0.09	0.153

Data of range of movement represent median and range; HCS, headless compression screw; RLP, radial head locking plate.

### Complications

In the HCS group, one patient underwent implant removal and radial head replacement at 7 months post-operatively due to non-union. Two cases showed 30° extension deficits with Hastings and Graham type IIA heterotopic ossification (HO), both managed with conservative treatment and physical rehabilitation. Two patients had posterior interosseous nerve injury with extensor digitorum weakness, resolving after conservative care. In one case, the screws was removed at 5 months post-operatively due to hardware irritation from excessive length.

In the RLP group, one patient underwent implant removal and radial head replacement at 9 months post-operatively due to non-union. One case had a 35° extension deficit (type IIA HO), two showed pronation limitations (type IIB HO), and all were treated conservatively. One severe case with approximately 43° pronation loss compared to the contralateral side underwent surgical implant removal and soft tissue release. Posterior interosseous nerve (PIN) injury was observed in three patients, presenting as extensor digitorum weakness. Among them, two resolved and one retained grade 4 muscle strength at the final follow-up. 13 cases required plate removal for hardware-related pain or skin irritation and the symptoms disappeared postoperatively. No wound infections were observed in either group during the follow-up period.

Though the incidence of minor complications showed no difference between the two groups (10.5% vs. 15.2%, *p* = 0.526), the HCS group demonstrated markedly lower rates of major complications requiring re-operation than the RLP group (5.3% vs. 32.6%, *p* = 0.002). Therefore, a statistically significant difference was observed in overall complication rates between the two groups.

The predominant indication for re-operation in the RLP group was symptomatic hardware irritation (2.6% vs. 28.3%, *p* = 0.002), whereas revision surgery, such as movement restriction and implant failure, showed no significant differences between groups (2.6% vs. 2.2%, *p* = 0.673, Table 5).

**Table 5:** Complications and reoperation indications at final follow-up.

Complication	HCS group *n* = 38	RLP group *n* = 46	RD (95% CI)	NNT (95% CI)	Unadjusted *P*-value	Adjusted *P*-value*
Total complication	6 (15.8%)	22 (47.8%)	−32.0% (−50.2, −13.8)	−3.1 (−7.2, −2.0)	0.002	0.012
Minor complication	4 (10.5%)	7 (15.2%)	−4.7% (−18.9%, 9.6%)	21.3 (NNTB 5.3 to ∞ to NNTH 10.5)	0.526	1.000
Major complication^a^	2 (5.3%)	15 (32.6%)	−27.3% (−42.6, −12.1)	−3.7 (−8.3, −2.4)	0.002	0.012
Re-operation	2 (5.3%)	15 (32.6%)	−27.3% (−42.6, −12.1)	−3.7 (−8.3, −2.4)	0.002	0.012
Hardware removal	1 (2.6%)	13 (28.3%)	−25.7% (−39.6, −11.8)	−3.9 (−8.5, −2.5)	0.002	0.012
Revision surgery	1 (2.6%)	2 (4.4%)	−1.8% (−10.0, 6.4)	−55.6 (NNTB 10 to ∞ to NNTH 15.6)	0.673	1.000

HCS, headless compression screw; RLP, radial head locking plate; RD, risk difference (negative value favors HCS); NNT, number needed to treat (negative NNT indicates the number needed to treat with HCS to avoid one adverse event compared to RLP; NNTB, number needed to treat for benefit; NNTH, number needed to treat for harm); CI, confidence interval.

a Primary pre-specified endpoint.

* Adjusted for multiple comparisons using the Bonferroni correction (6 comparisons; adjusted significance threshold = 0.0083). Adjusted *p*-values are calculated as unadjusted *p*-value × 6, capped at 1.000.

## Discussion

Radial head fractures represent one of the most common elbow injuries, predominantly caused by indirect axial loading transmitted through the forearm (12). The classification of radial head fractures has evolved since Mason introduced the original system in 1954, and Hotchkiss revised the classification in 1997 (4). The Mason classification system categorises these fractures into three types (Ⅰ-III) based on displacement severity and articular involvement, guiding treatment and rehabilitation strategies (4). Conservative management typically achieves favourable outcomes in Mason type I fractures (13). On the other hand, for Mason type III fractures, especially those that are comminuted and beyond reconstruction, arthroplasty is recommended. Mulders et al (3) have reported that nonoperatively treated adults with an isolated Mason type 2 radial head fracture with fragment displacement less than 2 mm and Involving less than 30% of the articular surface had similar functional results after 1 year compared with operatively treated patients. However, for Mason type II with with mechanical obstruction, surgical fixation with implants such as plates and screws is generally required (14, 15). Furthermore, severe comminuted radial head fractures with more than three fracture fragments are prone to early fixation failure and nonunion during fixation (16). Therefore, we excluded severe comminuted fractures with more than three fracture fragments.

Biomechanical analyses have shown no statistically significant difference in stiffness between plate and screw fixation for radial head fractures. Under longitudinal compression, the mean stiffness was 659.8 ± 29.4 N/mm for plates and 678.4 ± 117.0 N/mm for screws. Biomechanical analysis of plate vs. screw fixation for radial neck fractures with concomitant two-part radial head fractures demonstrated no statistically significant difference in stiffness under axial compression (17, 18). Studies by Li et al (19) and Smith et al (8) reported satisfactory outcomes (MEPS score >90, Broberg and Morrey score good or excellent) with both plate and screw fixation for radial head fractures. The efficacy of plates and screws in the treatment of radial head fractures was compared in the study by Yano et al (20). The results showed comparable efficacy of plate and screw in the treatment of radial head fractures and demonstrated a higher reoperation rate and complication rate in the RLP group. In our study, both groups achieved similar clinical outcomes, with comparable MEPS exceeding 90 points (*p* = 0.394) and no statistically significant differences in post-operative range of movement across flexion-extension (*p* = 0.577) or forearm rotation (*p* = 0.153–0.386).

In our study, the major complication and reoperation rate were lower in the HCS group than in the RLP group. In the RLP group, most patients who underwent secondary surgery did so not because of pain or implant failure, but due to long-term discomfort caused by foreign body friction during forearm movement. The symptoms disappeared after the removal of the internal plant. RLPs restore native radial head anatomy and enhance neck stability (21). The radial head was elliptical rather than perfectly round (22). Additionally, the shape, angle, and curvature of the proximal radius varied greatly (23). Thus, although anatomically contoured plates fit the radial head as closely as possible, the anatomical variations of the radial head make it difficult for the plates to completely match, which may be one reason for the increased complication rate. Moreover, the fixation area of the plates is limited to a safe zone, and improper placement can cause post-operative elbow joint mobility issues. Previous studies have shown that a larger dissection area during plate placement increases the rate of HO, thereby limiting rotation (8). Anatomical studies reveal that the PIN lies within 3.8 cm of the radial articular surface (24). This suggests that the exposure of the distal part of the radial head increases the risk of injury to the posterior interosseous nerve. Using HCSs reduces irritation of the internal fixator to the elbow joint and surrounding soft tissues while minimising the stripping of periosteum. Minimal distal soft tissue dissection optimally reduces the risk of PIN injury during surgical exposure.

This study had certain limitations. Firstly, when using screws to fix multiple fracture fragments, the possibility of screw interference must be considered, requiring higher surgical skills, which may affect the choice of the fixation method. Secondly, due to the low incidence of radial head fractures, a considerable timeframe was required to include cases that may affect follow-up assessments. The relatively limited number of enrolled patients reduces the statistical power to detect smaller but potentially clinically significant differences and precludes meaningful subgroup analyses. Thirdly, Due to the low incidence rate, this study required an extended follow-up period. This, in turn, increased the likelihood of data gaps and posed challenges in maintaining uniform data collection standards over the years. These factors may introduce unintended bias or confounding. Future multicentre, large-sample prospective cohort studies are required to provide further clinical evidence.

## Conclusion

For internal fixation of comminuted radial head and neck fractures with no more than three displaced fragments, both HCS and RLP achieved good outcomes. However, the RLP increased the incidence of complications and re-operation associated with internal fixation compared to HCS.

## Data Availability

The raw data supporting the conclusions of this article will be made available by the authors, without undue reservation.
